# Enterotype Variations of the Healthy Human Gut Microbiome in Different Geographical Regions

**DOI:** 10.6026/97320630014560

**Published:** 2018-12-29

**Authors:** Fauzul Mobeen, Vikas Sharma, Prakash Tulika

**Affiliations:** 1 School of Basic Sciences, Indian Institute of Technology Mandi, Kamand 175005, Mandi, Himachal Pradesh, India

**Keywords:** Enterotype, geographical factor, healthy human gut microbiome, inter-continental, inter-continental

## Abstract

Enterotypes are used for classifying individuals based on the gut microbiome. A number of studies are available to find the Enterotypes in healthy individuals; however, most of them lack comparisons at the world level. We analyzed the healthy human gut microbiomes of 495 datasets available in the European Nucleotide Archive (ENA) database derived from fifteen countries from four continents. Firmicutes and Bacteroidetes were the two most abundant phyla in the healthy human gut, worldwide. A high ratio of Proteobacteriato Actinobacteria and a low abundance of Prevotella were identified as the indicators of IBD. Prevotella, Bacteroides, and Bifidobacterium were identified as the Enterotypes in the inter-continental comparisons. At the intra-continental level, two (Bacteroides and Ruminococcaceae), four (Faecalibacterium, Bacteroides, Prevotella, and Clostridiales), and two (Prevotella, Bacteroides/Bifidobacterium) Enterotypes were identified in the American, European, and Asian continents, respectively. In addition, a high abundance of the unknown genus of Ruminococcaeae was observed in the Colombian human gut microbiome. A substantial impact of the geographical distance was observed on human gut microbiome variations, demonstrating a cumulative effect of factors, including dietary habits, genetics, lifestyle, environment, and climate, etc.

## Background

Healthy human harbors trillions of microbial cells with health promoting effects inside its gut, in a ratio of 1:1 with respect to the human cells 1,2, which is known as the "gut microbiome". The beneficial roles of the gut microbes have prompted a large number of studies towards exploring their roles in healthy individuals. Initial studies have focused on a limited number of healthy individuals from different regions and demonstrated differences in their gut microbiomes primarily due to inter-individual variations 3,4. With the advancements in the sequencing technologies, more studies, including the Human Microbiome Project (HMP) initiative 5 and the METAgenomics of the Human Intestinal Tract (MetaHIT) consortium project 6, have been initiated, which focused on a larger number of healthy human individuals and reconfirmed the inter-individual variations among the gut microbiomes. The compositional makeup of the gut microbiome is known to vary in healthy individuals, however, only to a very minute extent 7.

Due to the underlying inter-individual variations, healthy individuals can be clustered separately based on the differences in the enrichment of the microbial taxa in their guts 8. These enriched taxa are termed as Enterotypes and have been found to be independent of age, gender, body mass index(BMI), and geography. However, the methodologies and a few parameters used for determining the Enterotypes are known to affect the clustering of samples 9. For example, two independent studies conducted on the human gut microbiomes of healthy individuals from The United States of America (USA) demonstrated different numbers of Enterotype clusters 10,11. These differences could be attributed to the two different methods used for Enterotypedetermination in these studies. The other factor, viz., the variable region of 16S rRNA gene used for metagenomic sequencing is shown to moderately affect Enterotype-determination 9. The type of metagenomic sequencing, viz., 16S rRNA gene or Whole Genome Shotgun (WGS) based method 9, is known to weakly affect the Enterotype determination. In addition to being widely used as the potential biomarkers for healthy human gut, the Enterotypes have recently been used in context to predicting their associations with diseases 12 and dietary intervention 13.

An initial study conducted on the healthy individuals from the European countries, Japan, and USA demonstrated an existence of three Enterotypes in the healthy human gut microbiome 8. Another study conducted only on the individuals from the USA found only two Enterotype clusters 10. Further, the identified Enterotypes were not only limited to the originally identified taxa, viz., Bacteroides, Prevotella, and Ruminococcus, but Bifidobacterium and Enterococcaceae were also observed as Enterotypes in the individuals from Saudi Arabia and Taiwan, respectively 14,15. The Enterotypes have been determined at individual country level 14, intra-continental level 16, and also at inter-continental level 1[8]. Although, a few of these studies included a large number of samples in the Enterotype-determination at inter-continent level, however the numbers of countries included in these studies were limited 17,18.

In addition to the characterization of the Enterotypes, the impact of different factors, including age, geography, birth mode, antibiotics, diet, and genetics, on healthy human gut microbiome has been investigated 19. However, the effect of geography has been comparatively less studied 8, 20-21. In addition, these studies included only a limited number of countries ranging from one 22 to twelve 1[23] for their analyses. Of these, a human gut microbiome study on twelve countries demonstrated that latitude was positively and negatively correlated with the abundances of phyla Firmicutes and Bacteroidetes, respectively 23. In another study, the microbiome analysis of native Tibetan and Han Populations living at different altitudes showed a significant influence of altitude on the microbiome structure 22. Similarly, a recent study revealed geographical patterns of active and standing human gut microbiome in health and disease 24. Besides, there are a few studies which explored the effect of geographical location on the healthy human gut microbiome by using a combination of different microbial profiling methods 23. Interestingly, the gut microbial variation with respect to the geographical distance was not quantified in any of these studies.

In the present study, we have conducted a comprehensive Enterotype analysis by using publicly available 16S rRNA gene amplicon data from fifteen countries belonging to four continents, viz., Asia, Africa, America (North and South), and Europe. We have determined the compositional variation and diversity (alpha and beta) patterns present within the healthy human gut microbiomes with respect to their geographical locations followed by their quantifications.

## Methodology

### Data retrieval of publicly available human gut microbiome datasets:

The publicly available raw 16S rRNA gene based metagenomic datasets from fifteen countries, viz., Burkina Faso, Egypt, India, Malaysia, China, Japan, Taiwan, Thailand, Indonesia, Italy, Sweden, Spain, USA, Argentina, and Colombia were retrieved from the European Nucleotide Archive (ENA) (https://www.ebi.ac.uk/ena) (Table 2).

### Preprocessing of the datasets:

Paired-end sequences generated by the Illumina sequencing method were merged using the fastq-join method 25 with default parameters. The QIIME software package 26 was used for the downstream preprocessing analysis. After, the raw fastq sequences were converted into their corresponding fasta files and quality value files using python scripts available in the QIIME environment. This was followed by quality filtering process using the parameters, including, minimum length (150 bp), maximum length (1000 bp), and Q value (20). Further, the quality filtered reads were removed using the reference based chimera filtering method, usearch61 27, using the green genes database v13_8 (gg_13_8) 28. This was followed by the operational taxonomic unit (OTU) identification step, which was carried out by usearch61 method implemented in the vsearch tool 29 using the reference based OTU picking method against the gg13_8 database. The representative OTUs were used to elucidate the taxonomic inference of the reads by aligning them with the 16S rRNA gene sequences of the gg13_8 database using an identity cut-off of 97% using PyNAST 30. For calculating the ratio of taxa abundance at the phylum level (Firmicutes/Bacteroidetes and Proteobacteria/Actinobacteria) the country level relative abundances of the taxa, was used.

### Diversity analysis:

The alpha diversity of the datasets were calculated using three alpha diversity based methods, including Shannon index, Observed species, and Phylogenetic diversity (PD) whole tree. The t-test was employed for the determination of the significance among the alpha diversities of the countries. The microbial diversity among the datasets was estimated by calculating the beta diversity indices using weighted and unweighted unifrac distances. Further, a Principal Component Analysis (PCA) was carried out using the weighted and unweighted unifrac distances to calculate the similarity and dissimilarity among the datasets based on their microbiome structure and diversity. PCA results were visualized using the Emperor tool 31 present within the QIIME software. Further, jackknifed beta diversity was calculated using the unweighted and weighted unifrac distances to check the robustness of the beta diversity results.

### Enterotype determination:

To determine the Enterotype clusters in the human gut microbiome, a widely used enterotyping pipeline (http://enterotyping.embl.de) was used. The optimum number of the clusters was identified using the Calinski-Harabasz (CH) index, whereas robustness of the clusters were accessed using the Silhouette index. The Kruskal- Wallis test was implemented to check the significant differences (p < 0.05) of taxa abundance and diversity across Enterotypes.

### Core OTUanalysis:

The OTUs were considered as core OTUs of a given continent if they were present in at least 80% of the human gut microbiome samples with a relative abundance of at least 0.001 in any two countries of a given continent. Further, the shared and unique genera among the continents were calculated by only considering the presence or absence of the genera in the continents. To identify the shared genera at the inter-continental level, at first the presence of the given genus is confirmed in all the countries of a given continent. The genera from all the countries which qualified this stepwere further compared to identify those genera which were shared by all the continents. Similarly, unique genera were identified for the continents which were present uniquely in a given continent and was absent in all other continents.

### Correlation analysis between the geographical distance and human gut microbiome:

**Figure 1:**
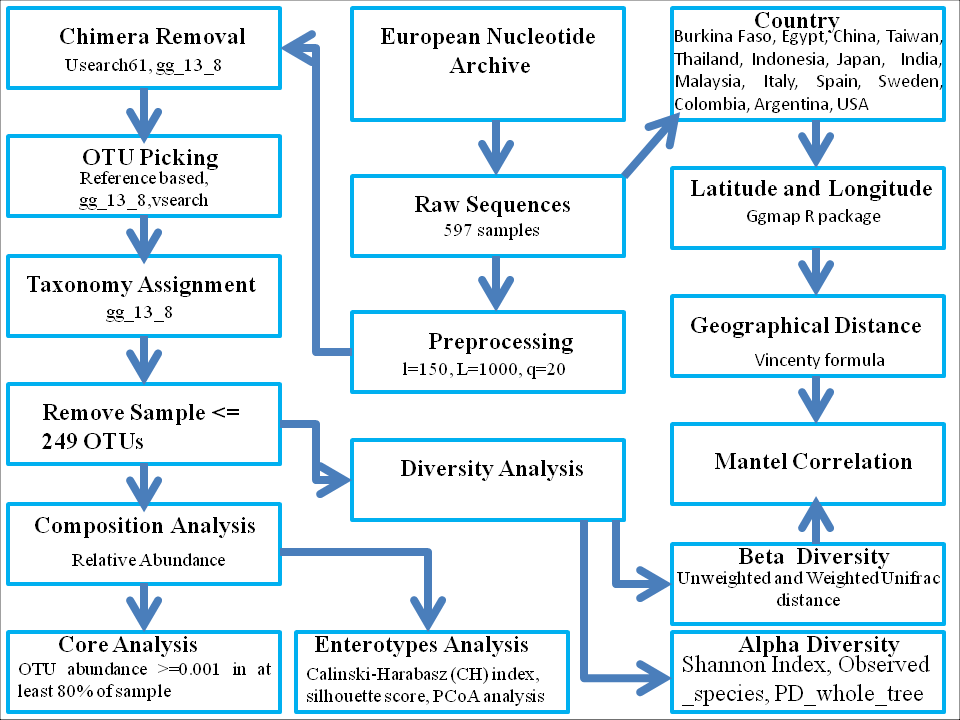
A flowchart of the methodology and bioinformatics tools used for the analyses.

The latitude and longitude of the countries analyzed in this study were retrieved using the ggmap R package 32. The geographical distance between the countries was calculated using the script available in the QIIME package. Further, a correlation between the gut microbiome distance (weighted and unweighted unifrac distance) and the geographical distance were calculated using the Mantel Correlation method 33. Mantel correlations were calculated between two distance matrices and the square of the Mantel correlation value explain the percent of variability present in one matrix due to the other matrix. The correlation between the latitude with bacterial (Firmicutes and Bacteroidetes) abundance was calculated using the Spearman's correlation. A p-value < 0.05 was considered for the statistical significance. A flowchart of methodology and bioinformatics tools used for the present analyses is given in Figure 1.

## Results and Discussion

We used 597 datasets representing healthy human gut microbiome from fifteen countries for this analysis. A total number of 31,126,235raw read sequences were obtained from these 597 datasets. The quality-filtering step resulted in a total number of 29,229,968reads. After removing the chimeric sequences, a total number of 28,427,739 read remained in the processed datasets. Following this, OTUs were predicted per sample and those samples containing low number of predicted OTUs (< 250 OTUs) were removed from the further analyses. This resulted in 495 datasets belonging to fifteen countries. In order to estimate the number of OTUs per country, all the OTUs predicted in each dataset of a country were combined together. The total number of predicted OTUs was found to vary from 1,257 to 5,660 ( Table 1).The sequencing depth also varied enormously among different countries, which is known to introduce biases during the estimation of rare taxa. In order to account for this bias, we performed a rarefaction analysis to estimate a minimum number of reads required to capture the complete diversity of each sample (Available with Authors) 1[34]. The sampling was found to be sufficient to capture the complete diversity of each dataset. Further, taxonomic assignments were carried out for the datasets of each country and the number of known genera identified in the samples varied from 57 to 152 (Table 1).

### Composition Analysis:

**Figure 2:**
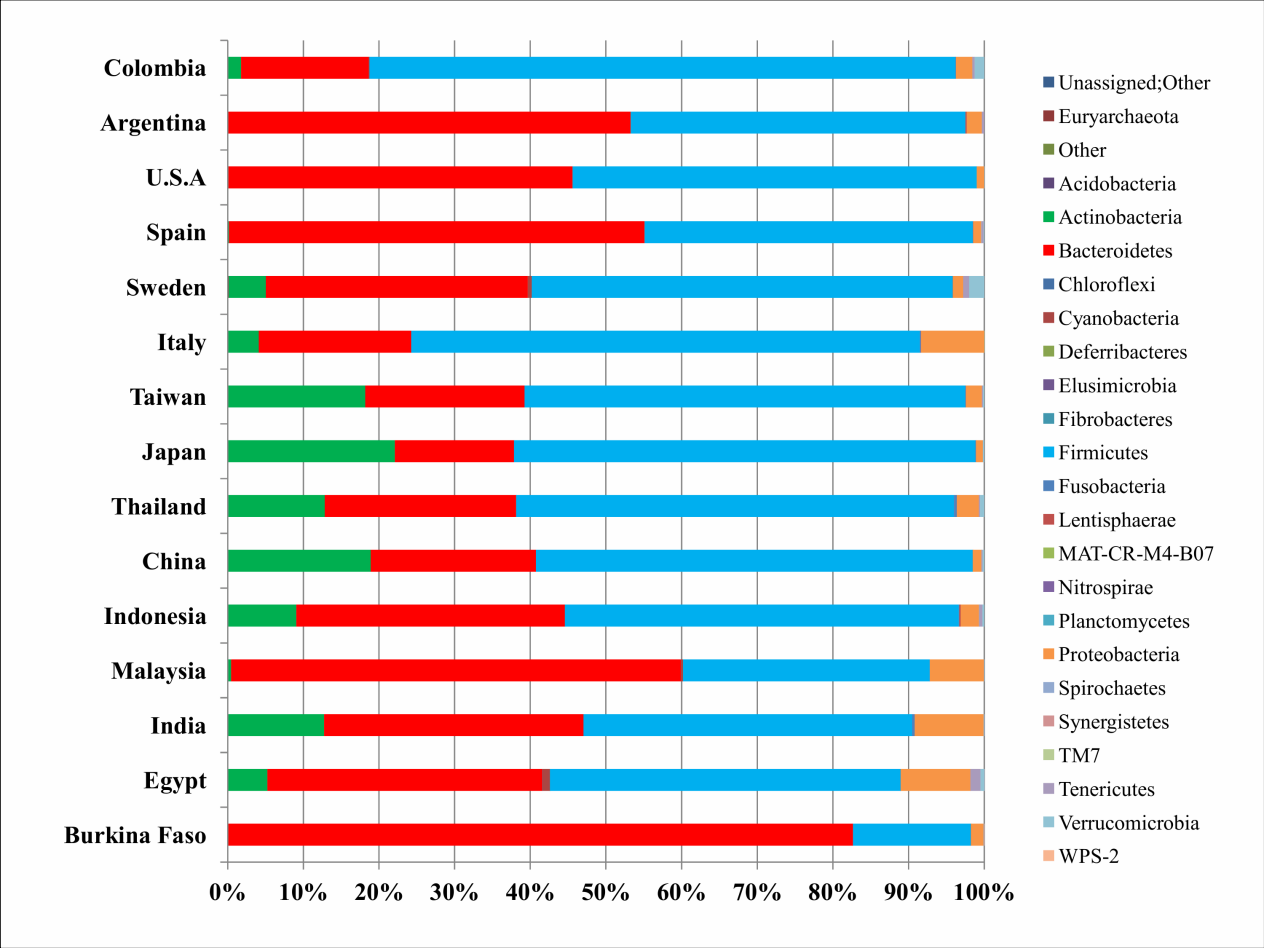
A bar plot of the relative abundance of different phyla identified in the gut microbiomes of fifteen countries. X axis shows the contribution (%) of each phylum and Y axis shows the countries used in this study.

In the present study, we have retrieved 24 phyla, 56 classes, 114 orders, 202 families, and 427 genera across all the 495 samples. Firmicutes and Bacteroidetes were found as the most abundant phyla (Figure 2), however, their relative abundances varied among different countries. At the country level, Colombian and Burkina Faso datasets were found to be at the extremes with Firmicutes and Bacteroidetes as the most enriched phyla, respectively. In order to determine the proportion of Firmicutes (F) to Bacteroidetes (B) in healthy human gut microbiome, we determined F/B ratio of the datasets from all the countries (Table 2). The F/B ratio was found to be > 1 for most of the Asian countries, except for Malaysia. Four out of six Western countries exhibited F/B ratio > 1. For those Western countries, which did not follow this trend, a very small difference was observed between the percentage abundances of these two phyla. We further observed positive and negative correlations of the relative abundances of the phyla Firmicutes and Bacteroidetes with latitude, respectively as observed previously 23, however, no statistical significance (p < 0.05) was observed (Available with Authors).

The phyla Actinobacteria and Proteobacteria were found to be the next major taxa in the healthy human gut microbiome (Figure 2). The phylum Actinobacteria is an important part of healthy human gut microbiome. Particularly, Bifidobacterium of Actinobacteriais of high relevance as it is one of the commonly used probiotics 35, because of its important roles in the immune system maintenance and protection against pathogens 36. Interestingly, Actinobacteria is the keystone taxon of microbiome due to its high degree of ecological connectedness to interact with other microbes 37. In our analysis, the relative abundance of Actinobacteria was found to be less in the human gut microbiome as compared to that of Firmicutes and Bacteroidetes and is consistent with previous studies 38.An overall higher abundance of phylum Actinobacteria was observed in the Asian continent as compared to that in the remaining three continents (Figure 2). Of all the Asian countries, Japan showed a highest relative abundance of this phylum, which is in corroboration with previous studies 39. Further analysis revealed Bifidobacterium as the most abundant genus of the phylum Actinobacteria in the Asian and the European countries (Available with Authors).

It is known that in the human gut, Bifidobacterium has high abundance of glycoside hydrolases to degrade starch than the other microbes residing in the gut 40. The populations of the Asian countries, including, Japan, China, and India, are known to consume high starch based diet on a regular basis. This might have possibly led to a high abundance of Bifidobacterium in the Asian gut microbiome as reported previously 14. Proteobacteria was found as the next most abundant phylum in our study in the healthy human gut microbiome after the phyla Firmicutes, Bacteroidetes, and Actinobacteria. In our analysis, Egyptian human gut microbiome showed the highest relative abundance of the phylum Proteobacteria followed by those of India, Malaysia, and Italy (Figure 2). The phylum Proteobacteria is known to significantly explain the existing variability of the human gut metagenome at the functional level 41.

### Alpha Diversity Analysis:

**Figure 3:**
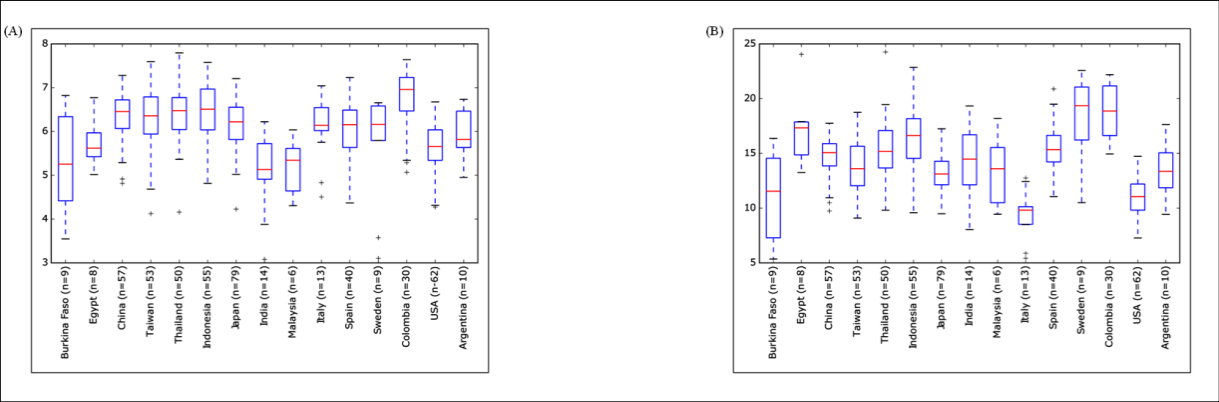
Alpha diversity box plots of the gut microbiome of the fifteen countries based on (A) Shannon index and (B) PD whole tree. X axis shows the name of the country and is arranged in continent wide order viz., Africa, Asia, Europe, and America along with the number of datasets, represented by n. Y axis shows the alpha diversity index.

The overall alpha diversity showed differences within the datasets of the different countries, but without any statistical significance (p < 0.05)(Figure 3A). Of these, the Shannon based alpha diversity, which represents the richness and evenness of the taxa, revealed a similar diversity pattern across the European countries. Within the Asian continent, also a similar pattern was observed, except for India and Malaysia, which exhibited very low alpha diversities in comparison to the other Asian countries. The African continent showed a high diversity in Egypt as compared to Burkina Faso. Further, Colombia exhibited a very high alpha diversity as compared to the other countries in the American continent. Among all the fifteen countries, Colombia and India exhibited the highest and the lowest diversities, respectively. This indicates Colombia to be the most and India to be the least diverse in the richness and evenness of the taxa.

The PD whole tree based index was used to infer the phylogenetic diversity of different datasetsto resolve the effect of phylogenetically related taxa on the overall alpha diversity. A lot of variation in the phylogenetic diversity was observed within the datasets of all the four continents, except for Asia (Figure 3B). Although all the European datasets exhibited a similar alpha diversity in terms of the evenness and richness of the taxa, however, differences were observed among their phylogenetic diversities. Maximum and minimum phylogenetic diversities were observed for Swedish and Italian datasets, respectively. However, the datasets of the American and African continents exhibited similar trends of the Shannon and Phylogenetic based alpha diversities. Among the Asian samples, only few minor differences were observed between the Shannon and Phylogenetic alpha diversities in different countries. Overall, Sweden exhibited the most phylogenetically diverse gut microbiome, whereas Italy was the least diverse in its gut microbiome.

### Beta Diversity Analysis:

**Figure 4:**
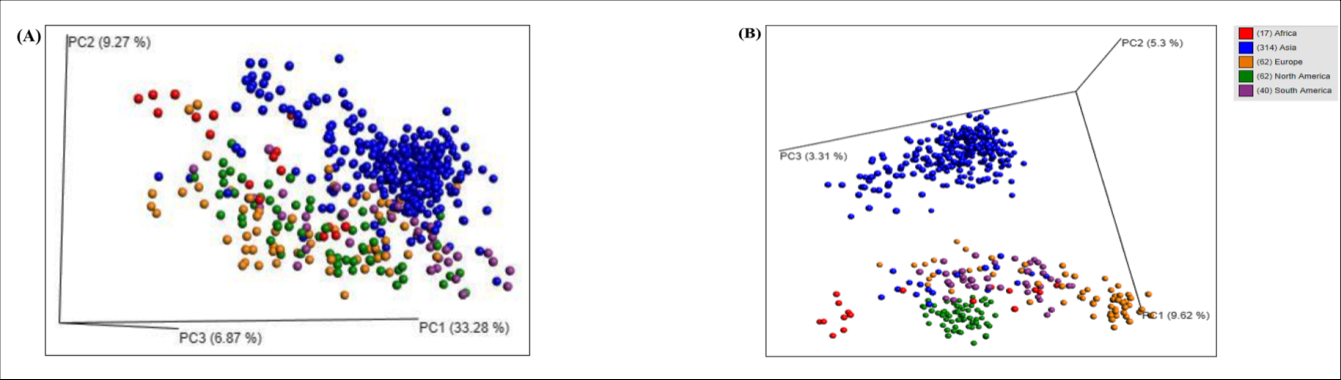
Beta-diversity based Principal Component Analysis (PCA) plots of the human gut microbiomes of fifteen countries using (A) weighted and (B) unweighted unifrac distances. X, Y, and Z axes show PC1, PC2, and PC3 components respectively. PC1, PC2, and PC3 axes explain 33.28%, 9.27%, and 6.87%, respectively, in (A) and 9.62%, 5.3%, and 3.31%, respectively, in (B) of the human gut microbiome variations present among the datasets.

In order to estimate the beta diversity, a PCA was performed on all the datasets using weighted unifrac distance metric (Figure 4A). This metric encompasses the effect of the abundance of taxa, which impacts robust clustering of the datasets than their separation into different clusters. Only the Asian datasets exhibited a distinct cluster along the PC1 and PC2 axes. A clear separation could not be obtained for all the other continents using this method. Hence, we opted for an unweighted approach as it is known to demonstrate an effect of only the presence and absence of the taxa on the separation of the gut microbial datasets in three-dimensional (3D) spaces. This analysis resulted in a clear separation of the samples belonging to the Asian, American, European, and African continents (Figure 4B). However, a mixed cluster was observed for a few datasets primarily belonging to the South American population followed by some datasets from the other countries, including, Egypt, India, Italy, Malaysia, and Sweden. It is interesting to note that although the Asian datasets differed from the samples of the other continents, however, they exhibited a large variation in the gut microbiomes among themselves, which is shown by the spread of the datasets along the PC2 axis. Similar findings were observed for the American datasets. Further, a better separation of the datasets into distinct clusters using the unweighted unifrac distance method indicated significant roles of the less abundant taxa for the distinction of the datasets.

### Enterotype Analysis:

**Figure 5:**
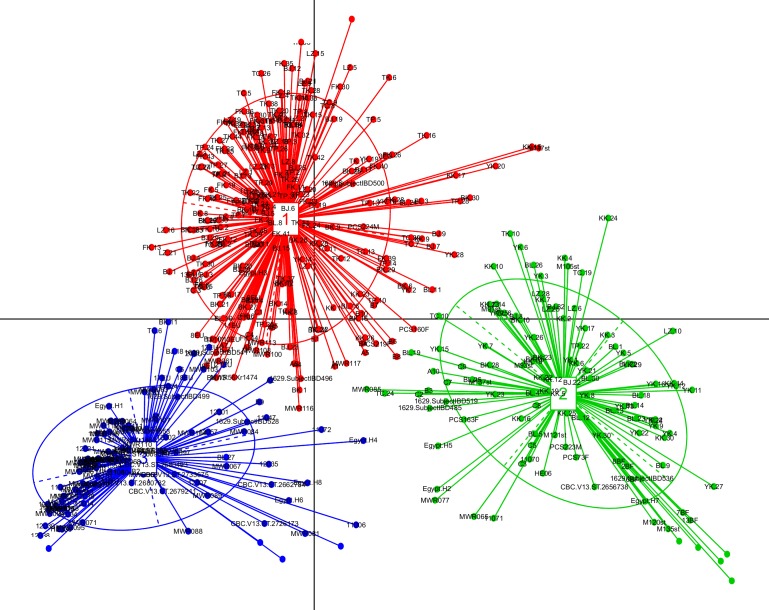
Visualization of three Enterotype clusters obtained from the Principal Coordinate Analysis (PCoA) identified in the healthy human gut microbiomes of 495 datasets from fifteen countries belonging to the four continents. The Enterotype taxa identified in cluster 1, cluster 2, and cluster 3 are Bifidobacterium, Prevotella, and Bacteroides, respectively.

**Figure 6:**
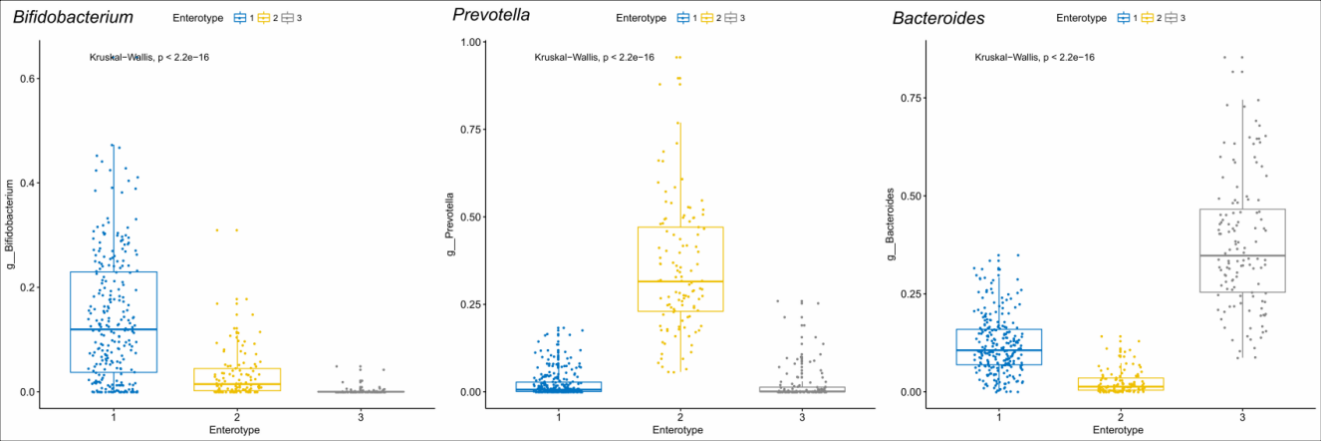
Box plots of the relative abundances of the Enterotype taxa in each of the Enterotype clusters identified in the healthy human gut microbiomes of 495. X axis shows the three identified clusters and Y axis shows the relative abundance of the taxa. The statistical significance for the difference in the relative abundances in each Enterotype cluster is calculated using the Kruskal-Wallis test (p less than 0.05).

We identified three optimal and robust Enterotype clusters using the Principle Coordinate Analysis (PCoA) from the gut microbiome of the 495 datasets (Figure 5). The genera Bifidobacterium, Prevotella, and Bacteroides were the highly abundant Enterotype taxa in the cluster 1, cluster 2, and cluster 3, respectively (Figure 6 (A - C)). A majority of the datasets from the Asian countries, including Japan, Taiwan, China, and Thailand were clustered into the Enterotype cluster 1 (Available with Authors). Thus, this cluster, dominated by the genus Bifidobacterium, might represent a signature taxon for the Asian-population. Cluster 1 also contained a few samples from some of the non-Asian countries, primarily including Colombia and Italy. The Enterotype cluster 2, which is mainly driven by the genusPrevotella, demonstrated a mixed cluster of various countries from different continents. A majority of the datasets from Burkina Faso, India, Indonesia, and Sweden were the members of this cluster. Further, a majority of the human gut microbiome datasets from Spain, Argentina, USA, Egypt, and Malaysia were clustered into the Enterotype cluster 3 with Bacteroides as the major driving genus for this cluster. Thus, this cluster might represent signature taxa for the Western-population.

**Figure 7:**
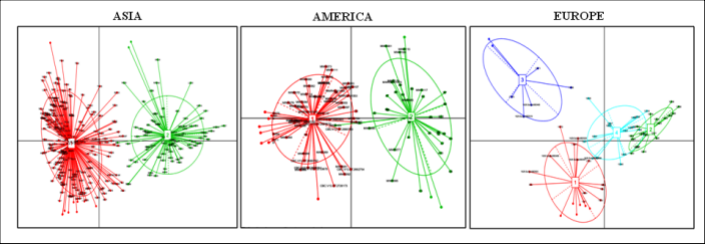
Visualization of the Enterotype clusters in Asian, American, and European datasets. The Enterotype taxa identified in clusters 1 and 2 of Asia are Bacteroides/Bifidobacterium (red) and Prevotella (green), respectively. The Enterotype taxa identified in clusters 1 and 2 of America are Bacteroides (red) and an unidentified genus of the family Ruminococcaceae (green), respectively. The Enterotype taxa identified in clusters 1, 2, 3, and 4 of Europe are Faecalibacterium (red),Bacteroides (green), Prevotella (blue), and an unidentified genus of the order Clostridiales (cyan), respectively. The Enterotype clusters in the human gut microbiome datasets have been identified using PCoA analysis.

Previously, Gorvitovskaia et al. had compared the worldwide population using a dataset of 747 healthy human individuals from the countries belonging to the four continents, viz., Asia, America, Europe, and Africa 18. They obtained two dominant Enterotypes, including Bacteroides and Prevotella. However, in our study, we also observed a third Enterotype cluster dominated by the Asian countries, represented by the genus Bifidobacterium. It is important to note that Gorvitovskaia et al. used only Japan as a representative country of the Asian continent, however,we have used seven Asian countries, including Japan, India, China, Taiwan, Indonesia, Thailand, and Malaysia. Earlier studies have demonstrated Bifidobacterium as a dominant Enterotype of Saudi Arabia [14]. The existence of this third Enterotype in our analysis could be mainly due to the enrichment of the genus Bifidobacterium in the Asian countries.In order to explore the geographic regional effects on the healthy human gut microbiome, we determined the Enterotype in each continent. We identified two, four, and two Enterotype clusters in the healthy human gut microbiomes of the American, European and Asian continents, respectively (Figure 7). For the African dataset, we found a very high number of the optimum clusters (Available with Authors)which may be due to a less number of the datasets included in our study, so the Enterotypes could not be determined. The Enterotype clusters identified in the Asian population were clearly separated into two clusters and exhibited no overlap. This indicated the occurrence of two sub-types of the Asian population based on the human gut microbiome. Enterotype cluster 1 of the Asian continent was dominated by the genus Prevotella (Available with Authors). This genus has been observed as one of the driving Enterotypes in the healthy human gutmicrobiomes ofthe Indian 42, Indonesian 16, and Kazakh populations 43. However, the relative abundances of two genera, including Bacteroides and Bifidobacterium were found to be high in cluster 2 (Available with Authors). This is in corroboration with a previous study, which was carried out using the Asian countries, including China, Japan, Taiwan, Thailand, and Indonesia 16. It is important to note that, we have included two more Asian countries, viz., India and Malaysia, in our analysis in addition to those included in the above mentioned study. Thus, the cluster 2 is driven by two dominant Enterotype genera viz., Bacteroides or Bifidobacterium. In a recent study, Enterobacteriaceae was identified as the third Enterotype in the Taiwanese population and was suggested as an Asian sub-Enterotype 15, however, we did not identify this taxon as an Enterotype.

The Enterotype clusters identified in the American continent were also clearly separated into two clusters and exhibited no overlap. This indicated the occurrence of two sub-types of the American population dominated by the Enterotypes Bacteroides and Ruminococcaceae. The datasets from the USA were distributed almost equally between these two Enterotype clusters. A few previous studies carried out on adults and children of the USA also demonstrated Bacteroides as the driving Enterotype of the healthy gut microbiome of this country 44,45. Most of the Argentinean datasets belonged to the Enterotype cluster Bacteroides, whereas, most of the Colombian datasets comprised a part of the Enterotype cluster driven by an unknown genus of Ruminococcaceae. This indicated that this unknown genus of Ruminococcaceae might be a potential Enterotype of the Colombian population. The Colombian gut microbiome exhibited very interesting results. In a very recent analysis with 441 Colombian human gut microbiome samples, no discrete Enterotype clusters of Bacteroides and Prevotella could be obtained [46]. A lack of Bacteroides and Prevotella Enterotypes in this population has been attributed to its gut microbial composition which is neither Western nor non-Western.

We also investigated the top ten highly abundant genera in each country (Available with Authors)and observed a high abundance of the members of the unknown genus of Ruminococcaeae in the Colombian human gut microbiome. The genus Bifidobacterium was absent in the top ten highly abundant genera in the Colombian datasets, thus confirming its very low abundance. Thus, it is an intriguing question that how a majority of the Colombian datasets still got clustered into the Enterotype cluster driven by the genus Bifidobacterium in our inter-continental analysis. The difference in the relative abundances of Bacteroides and Prevotella was found to be very low in the Colombian datasets as compared to those of the other countries. This might have prohibited the clustering of the Colombian datasets in the Enterotype clusters driven by these two genera and might have forced these samples to be a part of the third Enterotype cluster. A large dominance in the number of the samples from the Asian continent might be responsible for the formation of the third Enterotype cluster, which was primarily driven by the genus Bifidobacterium.

Unlike the Asian and American continents, the European continent was found to harbor four Enterotype clusters, which were dominated by the genera including, Faecalibacterium, Bacteroides, Prevotella and Clostridiales. Interestingly, the clusters belonging to the genera Bacteroides and Clostridiales were partly overlapping. This indicated that the European population could be further divided into three sub-populations based on the gut microbiome. Two of these sub-populations were dominated by the genera Faecalibacterium and Prevotella, while the third sub-population comprised a mixture of the individuals carrying the genera Bacteroides and Clostridiales as the dominant taxa. This also indicated that the European population is more diverse as compared to the Asian and American populations in terms of the dominating genera in their gut microbiomes.At the continent level, we could not detect any distinct Enterotype clusters in the African continent. However, during our intercontinental comparisons, a majority of the datasets from Burkina

Faso got classified into the Enterotype cluster driven by the genusPrevotella(Figure 5). The genus Prevotella has been identified as one of the driving Enterotype taxon in the adult rural African population in an earlier study 47. It is well known that the African diet consists of high fiber rich plants, which has been shown to increase the abundance of Prevotella in the gut microbiome 48. In our inter-continental comparison analysis, the majority of the Egyptian datasets exhibited Bacteroides and Prevotellaas the dominant Enterotype (Figure 5). In an earlier study carried out on healthy Egyptian children, the genus Bacteroides has been identified as the driving Enterotype taxon 49. However, this genus is also identified as the major driving Enterotype taxon in the Autism Spectrum Disorder and their neurotypical siblings in the Egyptian children 49. Interestingly, a recent study revealed the presence of Prevotella enriched Enterotypes clusters in the healthy Egyptian children [45]. Thus, the Enterotype status of the Egyptian population remains unclear and requires further investigation.

The members of the microbiome residing in the same niche are known to cross-talk among themselves, thus, might also affect the growth and survival of one another. In order to delineate the effect of the dominant driving taxa on the overall microbial diversity, we inferred the species richness in the Enterotype clusters. This analysis revealed a statistically significant lower microbial diversity (p-value < 0.05) only in the datasets belonging to the Enterotype cluster driven by Bacteroides (Available with Authors) which is in corroboration with an earlier study 12. A lower microbial diversity within the Bacteroides enriched human gut microbiome samples indicate a higher competition for the growth and survival of Bacteroides with the other microbes residing in the same niche. Prevalence/Indicators of Inflammatory Bowel Disorder: Inflammatory bowel disorder (IBD) is a very common gutassociated disorder throughout the world. A dysbiosis in the members of the phylum Proteobacteria has been linked to induce metabolic syndrome 50 and an overabundance of this phylum ascompared to that in healthy gut microbiome has been linked with IBD 51. On the other hand, a reduced relative abundance of Collinsella spp. of Actinobacteria has been implicated in IBD 52. This indicated that an increase and decrease in the relative abundances of the members of Proteobacteria and Actinobacteria, respectively, can be linked to IBD.

During the last century, IBD was more prevalent in the Western countries 53,however, recently it has been observed to increase in the Asian and African countries as well 54-56. Towards this, we explored the ratio of Proteobacteria (P) and Actinobacteria (A) in our samples (Table 2) (Available with Authors). Interestingly, P/A was found to be < 1 for all the Asian datasets with an exception of Malaysia. Recently it has been reported that the incidences of IBD are on a constant rise in Malaysia 56. All the western countries from the American and European continents, except for Sweden, exhibited a P/A ratio > 1. The other exceptions were Burkina Faso and Egypt, which exhibited P/A ratio >1, with Burkina Faso demonstrating the highest P/A value among all the fifteen countries. Recent studies showed an increase in the reporting of the IBD cases in the Egyptian and African population 55,57. Thus, the high P/A ratio as exists in the healthy human gut microbiome may be a factor associated with the increasing incidences of IBD. Another important indicator of predisposition towards IBD could be the enrichment level of the genus Prevotella in the healthy human gut microbiome. Recent investigation of the role of Prevotella Enterotypes in the healthy and disease gut microbiome revealed a less association of this genus with IBD 12. Thus, an enrichment of genus Prevotella in the human gut microbiome may confer beneficial health effects with respect to IBD. Interestingly, from our analysis, we observed a high enrichment of the genus Prevotella in the Asian and African countries (Available with Authors). A high abundance ofPrevotella has been linked with the dietary habits viz., intake of more plants and fiber based diets which are very common in Africa and Asia. However, the Western countries from the American and European continents, except for Sweden, exhibited a lower enrichment of the genus Prevotella.

Taken together, a higher P/A ratio and a lower abundance of Prevotella in the human gut microbiome might be the factors associated with the rising incidences of IBD. For example, the Western countries exhibited a higher P/A ratio and a lower abundance of Prevotella and are known to exhibit higher incidences of IBD. On the contrary, the Asian countries exhibited a lower P/A ratio and a higher abundance of Prevotella thus corroborating with comparatively lesser incidences of IBD. African countries exhibited a higher ratio of P/A and also a higher abundance of Prevotella. This indicated that these two factors have been counter-balancing each other and might be eventually promoting healthier phenotype. A shift in the trends of these factors might act as the indicator for the occurrence of IBD.

### Core Operational Taxonomic Units (OTUs):

Though the individual gut microbiomes differ from each other, there exists a core set of microbial members, which performs essential functions in the healthy human gut. Towards this, we identified 11, 98, 55, and 15 core OTUs in the African, Asian, European, and American continents, respectively (Available with Authors). In addition to the core OTUs in different continents, we identified a total of 115 genera which were present across all the four continents (Available with Authors). Although the continents share these common genera, however with varyingabundances (Available with Authors). A majority of the shared taxa were found to be belonging to the phyla Firmicutes, Bacteroidetes, Actinobacteria, and Proteobacteria. At the family level, a majority of the shared taxa across the continents were found to be belonging to Lachnospiraceae, Coriobacteriaceae, Veillonellaceae, Enterobacteriaceae, Ruminococcaceae, and Erysipelotrichaceae.

The functional roles of the core taxa were analysed comprehensively by performing thorough literature survey (Available with Authors).The members of the family Lachnospiraceae are the known producers of short chain fatty acids (SCFAs), including acetate, butyrate, and propionate by the degradation of the complex polysaccharides. These SCFAs are further utilized by the host for their energy needs. The members of the family Ruminococcaceae are well known butyrate producers of butyrate from carbohydrate. Some members of the family Veillonellaceae have been known to produce propionate from lactate. Coriobacteriaceae family is found to be implicated in the bile acid metabolism processes. Further, recent studies indicated the association of family Erysipelotrichaceae with the lipid metabolism of host. These results indicate that the healthy human gut microbiome consists of the common microbial members which are mainly responsible for fulfilling the metabolism and energy requirements of the host. In addition to the presence of the common genera across the continents, we also observed 58, 70, 10, and 25 unique genera, which were found to be exclusively present in the continents, Asia, Africa, Europe, and America, respectively (Available with Authors).

### Correlation between the Geographic Distance and the Microbiome Variation:

We have investigated the effect of the overall geographical distance of the fifteen countries on the healthy human gut microbiome variations based on the weighted and unweighted unifrac distance metrics. The weighted unifrac distance reflects the effect of the abundance of the microbial taxa. A Mantel correlation value of 0.34 (Rw) was observed between the geographical distance and the weighted unifrac distance matrix. This implied that 11% (Rw2) of the healthy gut microbiome variations based on the abundant taxa might be explained by the geographical distance. A higher Mantel correlation value of 0.64 (Ru) was observed between the geographical distance and the unweighted unifrac distance matrix.

It is important to note that the unweighted unifrac distance reflects the effect of only the presence and absence of the microbial taxa and is independent of the relative abundance of the taxa. This implied that 40% (Ru2) of the gut microbiome variations based on the taxa present in gut, including rare taxa, might be explained by the geographical distance. These observations indicate that the geographical variations might be affecting the diversities of the rarer abundant taxa more than these of the highly abundant taxa.

## Conclusion

The present study has demonstrated significant differences among the healthy human gut microbiomes of different countries, in terms of the composition, diversity, and Enterotypes. Three dominant Enterotype clusters driven by the taxa Bifidobacterium,Prevotella, andBacteroideswere identified across the worldwide population. The generaBifidobacteriumand Bacteroides were identified as the major Enterotype taxa of the Asian and Western (American and European) populations, respectively, whereas, the African datasets were distributed between Prevotella and Bacteroides. The Enterotype clusters driven by the genera Bacteroides and Prevotella were more robust as compared to the third cluster. The composition of the third cluster might vary with respect to the datasets included in the analysis. The members of the family Ruminococcaceae were identified as the signature taxa of the Colombian population.Besides these distinctions in the gut microbiomes, core taxa conserved across the continents were identified with predominant involvements in the process of metabolism and energy production.The low abundant taxa were identified as the important factors for the distinction of the microbiomes from different geographical regions. Such, low abundant taxa were also found to be predominantly affected by the geographical distances. In addition, a higher P/A ratio and a lower abundance of Prevotella might be an indicator of the rising incidence of IBD, however, comprehensive studies on IBD patients are required to confirm this hypothesis. It is important to note that the possibility of our observations being affected due to the differences in the variable regions of the 16S rRNA gene used in the independent studies, cannot be ruled out. This analysis provides a snapshot of the effect of the geographical factor on the healthy human gut microbiome which may be impacted by various other factors, including dietary habits, genetics, lifestyle, environment, and climate, etc.

## Figures and Tables

**Table 1:** Summary of the 16S ribosomal RNA gene sequence based metagenomic analysis results of the datasets used in this study.

Country	Number of samples used in this study*	Average read length	Number of reads used in this study^	Average number of reads per sample	Number of OTUs	Number of genera	No of unknown OTUs	Reads mapping to known genera (%)	Reads mapping to unknown genera (%)	ENA Accession Numbers	References
Burkina Faso	9	254	173012	19223.56	1257	57	26	91.93039	8.06961	ERP000133	[20]
Egypt	8	253	383613	47951.63	3368	121	81	64.55591	35.44409	PRJNA328966	[58]
China	57	402	245706	4310.632	3551	94	26	70.46069	29.53931	PRJDB1664	[16]
Taiwan	53	402	269446	5083.887	3509	89	24	71.48267	28.51733	PRJDB1664	[16]
Thailand	50	400	326087	6521.74	3922	108	34	71.77727	28.22273	PRJDB1664	[16]
Indonesia	55	401	354766	6450.291	3961	117	39	75.82541	24.17459	PRJDB1664	[16]
Japan	79	403	457678	5793.392	3572	85	29	67.03656	32.96344	PRJDB1664	[16]
India	14	175	15072369	1076598	5660	152	49	76.8114	23.1886	SRP041693, SRP055407, DRA002238	[59]
Malaysia	6	186	4196544	699424	4287	105	34	78.07753	21.92247	SRP079939	[60]
Italy	13	262	205151	15780.85	2347	73	21	57.50687	42.49313	ERP000133	[20]
Spain	40	448	1479424	36985.6	3693	101	37	66.12199	33.87802	PRJNA350839	[61]
Sweden	9	98	2531749	281305.4	5085	95	34	61.0206	38.9794	ERP020401	[62]
Colombia	30	217	513077	17102.57	4030	102	41	53.73231	46.26769	ERP003466	[39]
U.S.A	62	512	2122144	34228.13	4207	107	38	68.82446	31.17554	PRJNA297510	[63]
Argentina	10	483	88490	8849	1470	58	25	69.30452	30.69548	SRP062999	[44]
	495		13346887		53919						

**Table 2:** Relative abundance of the four major phyla in the fifteen countries. F/B ratio is calculated by dividing the relative abundance of the phylum Firmicutes by that of the phylum Bacteroidetes. P/A ratio is calculated by dividing the relative abundance of the phylum Proteobacteria by that of the phylum Actinobacteria.

Country	Actinobacteria (A)	Bacteroidetes (B)	Firmicutes (F)	Proteobacteria (P)	F:B Ratio	P:A Ratio
Burkina Faso	0.00044	0.8259175	0.156003	0.01687391	0.1889	38.1818182
Egypt	0.05142	0.3636299	0.463215	0.09218764	1.2739	1.79292686
India	0.12755	0.3427601	0.434293	0.09111048	1.267	0.71432678
Malaysia	0.00452	0.5944239	0.326254	0.07135572	0.5489	15.7759574
Indonesia	0.09018	0.3543298	0.520296	0.02418343	1.4684	0.268176
China	0.18899	0.2185131	0.577489	0.01138291	2.6428	0.0602317
Thailand	0.12737	0.2527141	0.578405	0.0289495	2.2888	0.22729082
Japan	0.22107	0.1572951	0.609805	0.00917998	3.8768	0.04152541
Taiwan	0.18171	0.2106475	0.582618	0.02100197	2.7658	0.11557767
Italy	0.041	0.2016742	0.672442	0.08360664	3.3343	2.03922576
Sweden	0.04879	0.3461723	0.556367	0.01356896	1.6072	0.27813482
Spain	0.00076	0.5489722	0.434036	0.01024094	0.7906	13.421859
U.S.A	0.00108	0.4545857	0.53414	0.00981369	1.175	9.08569807
Argentina	0.00054	0.5316702	0.442376	0.02003782	0.832	37.2580645
Colombia	0.01751	0.1683911	0.774891	0.02181417	4.6017	1.24605164
